# Orangutans and chimpanzees produce morphologically varied laugh faces in response to the age and sex of their social partners

**DOI:** 10.1038/s41598-024-74089-x

**Published:** 2024-11-06

**Authors:** Fabio Crepaldi, Florence Rocque, Guillaume Dezecache, Leanne Proops, Marina Davila-Ross

**Affiliations:** 1 School of Psychology, Sport and Health Sciences, University of Portsmouth, Portsmouth, UK; 2 College of Arts and Law, University of Birmingham, Birmingham, UK; 3 LAPSCO, Université Clermont Auvergne, CNRS, Clermont-Ferrand, France

**Keywords:** Orangutans, Chimpanzees, Laugh faces, Open-mouth faces, Social play, Smile, Behavioural ecology, Evolutionary ecology

## Abstract

Laugh faces of humans play a key role in everyday social interactions as a pervasive tool of communication across contexts. Humans often vary the degree of mouth opening and teeth exposure when producing these facial expressions, which may depend on who their social partner is (e.g., their gender and age as well as their social relationship), serving this way different functions. Although it was found that laugh faces show evolutionary continuity across humans and non-human great apes according to the Principle of Maximum Parsimony, little is known about the function of laugh face variations from an evolutionary perspective. Hence, the present work examined the morphology of laugh faces in orangutan and chimpanzee dyadic play to test if they are modified with dependence on the playmate’s characteristics (sex, age and social relationship). In total, we analysed over 600 facial expressions of 14 orangutans and 17 chimpanzees by coding the specific muscle activations (Action Units, i.e. AUs) contributing to these expressions, using OrangFACS and ChimpFACS, respectively. Our results suggest that age difference and, to a lesser extent, playmate sex influence laugh face morphology in both taxa, but in opposite ways. While the orangutans of our study seem to expose their upper teeth (with AU10) and to pull the mouth corners (with AU12) more towards weaker partners (younger and female), possibly to communicate non-hostility, the chimpanzees showed both upper and lower teeth exposure (with AU10 and AU16) more often when interacting with the stronger partners (older individuals), possibly to communicate submissiveness. These findings suggest that the ability of humans to modify laugh faces with dependence on social partner characteristics has most likely evolved from pre-existing traits, going back at least to the last common ancestor of today’s great apes, including humans.

## Introduction

Laughter and smiles are pervasive expressions of human social interactions^1,2^. Some may be mere outbursts of positive arousal, while others may be more communicative behaviours with different social functions, including affiliative and submissive ones^3–5^. The modulation and function of these behaviours may depend on several factors, such as the characteristics of the social partner^6^, including their relationship with them, as well as the context of the interaction^7^. For instance, laughter and smiles seem to be more addressed towards friends than strangers as a function of affiliation^2,8^. Forced/polite smiles, with no teeth exposure, are directed more towards males compared to females, and towards older individuals compared to younger playmates as a function of submissiveness or politeness^9–14^. Conversely, open smiles (with teeth exposure) are produced by young adults more often towards females than males and, in general, more towards peers than younger or older individuals^10,11^. Such sex-driven differences may be predominant among members of the same social status rather than among members of a lower or higher one^10^.

These differences in form and function of human smiles seem to develop early^15^. Already infants seem to produce broad smiles with mouth opening and teeth exposure more when their mother approaches and closed smiles with no teeth exposure more when a stranger approaches^16^. Infants also seem to produce more broad smiles when playing with their father instead of their mother, a pattern which might be explained by the fathers preferring to play rough-and-tumble^17^. Related to this, infants and pre-school children were found to display more closed smiles when playing alone, with partly exposed teeth during social interactions such as in gentle play, and with more extensive teeth exposure during mock-fight play^18^.

Evolutionary reconstructions of laughter and smiles in both form and function may help us better understand the complexity of these pervasive human behaviours as well as their role in the evolution of human emotions and cognition. The systematic approach of measuring facial muscle movements via the FACS-based tools, originally developed to identify such activations (Action Units, AU) in humans^19^ and later adapted to examine nonhuman species, including orangutans (OrangFACS^20^) and chimpanzees (ChimpFACS^21^), can be very helpful for such a task. A study by Davila-Ross and colleagues^22^ examined laughing in chimpanzees during spontaneous social play and found that these chimpanzees showed the same facial muscle activations when vocally laughing as when humans are laughing^23^. Based on the Principle of Maximum Parsimony, where simplest explanation is generated for phylogenetic reconstructions since evolution is unlikely to abolish and rebuild traits of comparable function among related species, it seems reasonable to conclude that these open-mouth laugh faces of the chimpanzees (and of other nonhuman apes) and the open-mouth faces of laughing humans (open-mouth smiles) are homologues (for a more detailed explanation of the Complexity and Continuity hypothesis of laughter and smiles, see^24^). Thus, the human open-mouth smile (i.e., laugh face), similar to human vocal laughter^25^, most likely was produced by ancestral apes, going back at least 10–16 million years^22,25^.

To this date, however, little is known about the evolutionary functions of great ape laugh faces. While their laugh faces may be linked to positive affect and high arousal, they may additionally have a communicative function to signal “this is play” (similar to the open-mouth faces of play in other mammals^26,27^). Such communication is notably important during rough play, which increases the risk of escalation into a real fight, and may be especially pertinent when there is an asymmetry in the strength of the individuals^28–30^. Signalling playfulness may, thus, be useful for individuals both to avoid getting hurt by a stronger partner and to reassure a weaker partner, who might otherwise stop playing for self-preservation. In great apes, laugh faces seem to occur particularly often during high intensity play^30–34^, especially in juveniles and adolescents^35,36^ and seemingly also in males, perhaps due to the rougher play style compared to females^37–40^.

It remains unclear though if there are variants of these expressions that are used as submissive and/or reassuring signals in nonhuman primates, like in humans. To better understand such complex relationship in both form and function of great ape laugh faces, we need to, therefore, investigate to what extent the use of variants of these expressions might be affected by the playmate characteristics, e.g. their sex, age and their social relationship. To our knowledge, the only finding on great apes linking form of laugh faces with social partner characteristics is a study on juvenile lowland gorillas, where they were found to expose their upper teeth more when playing with individuals of similar size/strength^40^. Such similarity in physical strength between these individuals makes it difficult to determine if the expression constitutes a submissive signal or a reassuring one or neither.

A slightly different pattern has been observed in crested macaques (*Macaca nigra*) when examining facial expressions with teeth exposure (bared-teeth displays) across behavioural contexts. Here the expressions were found to be more intense (i.e. with more highly activated facial muscles) in dyads with little difference in dominance rank between the individuals compared to dyads where it was higher^41^, possibly to reduce uncertainty and, therefore, misunderstanding. Adult geladas (*Theropithecus gelada)*, instead, were found to show more exaggerated open-mouth faces, with both rows of teeth exposed, than their younger playmates during social play^42^. However, while immatures used this play face’s form more when playing with older individuals compared to peers, suggesting a submissive function, adults produced it equally in adult-juvenile and in adult-adult dyads^42^. It is thus difficult at this stage to understand the function of facial variants in nonhuman primate play, a topic that, as this literature review showed, has been poorly investigated outside our species.

There is, however, evidence in great apes revealing that laugh faces may be present more often with dependence on the social partner. Specifically, in bonobos (*Pan paniscus*) and lowland gorillas (*Gorilla gorilla gorilla*), laugh faces seemed to occur differently with dependence on the partner’s sex, but in different ways. The bonobos produced laugh faces predominantly when in female-female dyads (compared to mixed-sex ones)^43^, while the gorillas produced them predominantly when in male-male dyads (compared to mixed-sex or female-female)^40^. Furthermore, bonobos produced laugh faces more frequently among individuals closer in terms of rank or age (and therefore size)^43,44^. In chimpanzees, no such differences were found^44^.

Laugh faces of great apes have been identified with OrangFACS^26^ and ChimpFACS^21^ as open-mouth displays that have the lips parted (Action Unit 25) and the mouth open, at least to the extent of a jaw drop (AU26)^24^. Furthermore, great ape laugh faces are frequently characterised by a combination of four distinctive facial muscle activations^22,45^: upper lip raising (AU10), lower lip depressing (AU16), lip corner pulling (AU12), and mouth stretching (AU27). AU10 exposes the upper teeth, AU16 exposes the lower teeth, AU12 enhances the teeth exposure when already present, and AU27 widens the mouth even further, replacing the jaw drop (AU26).

These four AUs are, therefore, the most likely to contribute to signalling playfulness when produced as part of the laugh face (also see Refs.^28,46^). Such signalling is perhaps clearest when it comes to the lower teeth exposure (AU16) as seen in the relaxed open-mouth display^47^ and the stretching of the mouth^48^, which have been linked with prototypical expressions in play (and more distinct from other open-mouth expressions outside of play; see the Power Asymmetry Hypothesis^49^). In the context of play, the upper teeth exposure, primarily driven by AU10 and AU12, has also been linked to play behaviours, especially rough play^30,44^, although some researchers have also proposed a connection with arousal^30^. In light of the facial variations of laugh faces based on social circumstances, the aforementioned four AUs are arguably crucial when investigating the function of such morphological variants.

The aim of the present work, therefore, was to examine the relationship between laugh face morphology and playmate characteristics in orangutans and chimpanzees, the great apes evolutionarily furthest and closest (together with bonobos) to humans, respectively^50^. We hypothesized that these great apes modify their laugh face morphology with dependence on their playmate’s characteristics, i.e., sex, age difference and social relationship, similar to humans.

We used OrangFACS^20^ and ChimpFACS^21^ to measure the underlying muscle activations of the laugh faces in the two taxa. We defined laugh faces by the parting of the lips (AU25) and a mouth opening at least to a jaw drop (AU26)^24^. To test for the potential impact of playmate characteristics, we included the four AUs that were predominant in orangutan and chimpanzee laugh faces^22,45^, i.e., AU10, AU12, AU16 and AU27, which are likely to signal playfulness. Given the mixed findings from previous research, we formulated two competing predictions: the great apes produce the predominant laugh face AUs more often when their playmates are stronger (e.g. older or male) and socially more distanced (Prediction 1) vs. the great apes produce the predominant laugh face AUs more often when their playmates are weaker (e.g., younger or female) and socially closer (Prediction 2).

We also tested for potential confounds in form of play intensity, biting and facing each other^24,51^ as it is possible that they occur differently with dependence on the playmate characteristics (e.g., playing less or more roughly when the playmate is stronger or a male, respectively). Such changes in behaviours could affect facial muscle activations in laugh faces, especially as facial muscle activations were previously found to change as a result of the presence of biting behaviour, of facing the playmate and of rough play in great apes^30,33,45,51,52^. For instance, orangutans were found to produce more complex (with more AUs involved) and marked (with a wider open mouth) laugh faces when facing the playmate compared to when the face is not visible to the social partner^45^. Furthermore, gorillas and bonobos were found to show more their upper teeth in rougher play contexts^30,40,44^.

## Methods

### Subjects and study sites

For the orangutans, the subjects (the individuals producing the laugh face) were 14 rehabilitant individuals (8 males and 6 females) ranging from 4 to 16 years old (mean = 8.00 years; SD = 3.09). Their playmates (the individuals playing with the subjects) were 17 individuals (the 14 subjects plus three orangutans, one male and two females, mean = 8.00 years) with the same age range. For an overview of the number of subjects, playmates, their sexes and their age group, see Table 1. The orangutans of this study lived at the Sepilok Orangutan Rehabilitation Centre (SORC) in Sabah, Malaysia. Supplementary food was givenat SORC twice a day, at approximately 10am and 3 pm, in two outdoor locations, respectively. The orangutans tended to socialise and interact during these feeding sessions and, apart from three mothers with infants, there were no related individuals roaming the SORC area. SORC’s rehabilitation programme included three stages. At stage 1, the infants were cared for by staff and volunteers and kept in a separate area, where they had access to the surrounding forest for a limited time. At stage 2, the juveniles had unlimited access to the forest during the day, but slept indoors during the night; they were given supplementary food. At stage 3, juveniles and mature orangutans were free-ranging in the forest and had no access to the buildings; they had the option to feed on supplementary food or to obtain food from the surrounding Kabili-Sepilok Forest Reserve.

**Table 1:** Details on the subjects and playmates of this study.

Subjects		Playmates		N dyads	N faces
Orangutans					
Juvenile	Female	Juvenile	Female	2	7
			Male	13	41
		Mature	Female	1	1
			Male	6	23
	Male	Juvenile	Female	15	34
			Male	15	41
		Mature	Female	9	23
			Male	14	35
Mature	Female	Juvenile	Female	2	5
			Male	7	14
		Mature	Female	0	0
			Male	4	8
	Male	Juvenile	Female	5	14
			Male	12	28
		Mature	Female	4	7
			Male	6	21
Total	14	17		115	302
Chimpanzees					
Infant	Female	Infant	Male	2	7
		Juvenile	Female	2	5
		Mature	Female	2	6
			Male	5	11
	Male	Infant	Female	2	8
		Juvenile	Female	2	57
		Mature	Female	3	12
			Male	3	3
Juvenile	Female	Infant	Female	0	0
			Male	2	39
		Juvenile	Female	2	36
		Mature	Female	5	35
			Male	5	11
Mature	Female	Infant	Female	1	7
			Male	2	2
		Juvenile	Female	5	26
		Mature	Female	0	0
			Male	5	13
	Male	Infant	Female	3	9
			Male	1	1
		Juvenile	Female	6	8
		Mature	Female	6	13
			Male	7	13
Total	17	18		71	322

Sex and age class are presented, along with the number of dyads and laugh faces obtained for each dyadic combination. Infants were 4 years or below, juveniles were older than 4 and up to 8, and mature (adolescents and adults) were older than 8. Four individuals (three orangutans and one chimpanzee) were included as playmates only because they engaged in play in less than 30 focal videos.

For the chimpanzees, the subjects were 17 individuals (8 males and 9 females), ranging from 1 to 36 years old (mean = 14.29 years; SD = 12.08). Their playmates were 18 individuals (the 17 subjects plus one female; mean = 14.72 years) with the same age range. They were semi-wild and lived in a 190-hectares enclosure (enclosure 1) of the Chimfunshi Wildlife Orphanage (CWO) in Zambia. Their multiple-family social group was stable and consisted of 24 individuals (8 males and 16 females) of different ages: 5 immature and 19 mature chimpanzees. For an overview of the number of subjects and playmates and their sex and age groups, see Table 1. The individuals of 26 years or younger are sanctuary-born, while the older chimpanzees were rescued from illegal trades during their first years of life. These chimpanzees were fed two times a day (at approximately 11am and 2 pm) by staff. Some ate inside a building attached to the outdoor enclosure and they then stayed indoors throughout the time in between the two feeding sessions. The chimpanzees tended to socialise and interact in the outdoor feeding area before and after the feeding sessions (where they were visible to researchers), but they stayed in the inner part of the enclosure most of the day (where they were not visible to the researchers).

### Video recording collection

For orangutans and chimpanzees, approximately 52 and 83 h of video recordings (focal + ad libitum) were examined, respectively. Focal recordings^53^ were taken twice daily (around the morning and afternoon feeding sessions) in a randomised order and consisted of 3- and 5-min for the orangutans and chimpanzees, respectively. Ad libitum recordings consisted mainly of play sessions and feeding, without following any specific subject order nor having a predetermined recording length. The videos were taken at two to approximately twenty metres. The orangutan videos were recorded from April 2016 to July 2017 (except April 2017) by six researchers, including FC, via a Panasonic HC V250EB-K Full HD camcorder (Panasonic, London, UK). The chimpanzee videos were recorded from May to September 2017 by two researchers via Sony HandyCam DCR-TRV19E (Sony Electronics, Oradell, NJ, USA). All video recordings took place outdoors.

The video recording was approved by the Sabah Biodiversity Centre (for SORC) and by the Chimfunshi Research Advisory Board (for CWO). The research ethics applications were approved by the University of Portsmouth’s Animal Welfare and Ethical Review Body (orangutan reference number: 816C; chimpanzee reference number: 1216A) and the methodologies were carried out in accordance with the ARRIVE guidelines^54^.

### Behavioural coding

We searched for dyadic play bouts throughout the available video recordings. Play bouts were identified as dyadic interactions which started when at least one individual produced a play action and ended when at least one individual withdrew for at least 20 s or when a third individual/human interfered^32,55^. In total, we identified 115 and 71 different subject-playmate dyads for the orangutans and chimpanzees, respectively. For an overview of the identified dyads and their number of laugh faces in orangutans and chimpanzees, see Table 1.

**Fig. 1:**
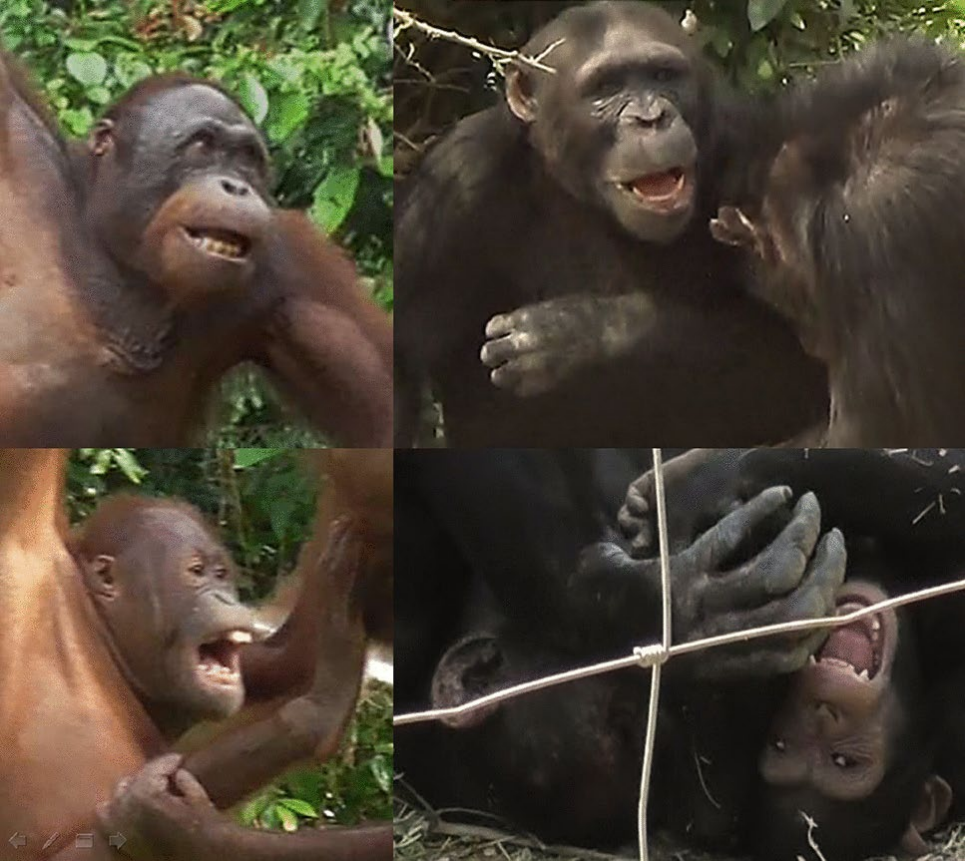
Examples of laugh faces in orangutans (left) and chimpanzees (right). First row: lip parting (AU25), jaw dropping (AU26) and lower lip depressor (AU16); second row: lip parting (AU25), mouth stretch (AU27), upper lip raiser (AU10) and lower lip depressor (AU16). For a scheme of the AUs typically involved in a laugh face see Davila-Ross and colleagues^22^, and for more details about AUs in these taxa, see the OrangFACS and ChimpFACS manuals^20,34^.

We then searched for laugh faces within each play bout. Laugh faces were defined as open-mouth displays that showed the lips parted (Action Unit 25, based on FACS^19^) and the jaw dropped (AU26) as a minimal requisite, whereby the jaw drop could increase in intensity and be replaced by a mouth stretch (AU27) (see Fig. 1 for examples of laugh faces). Laugh faces ended when the mouth was closed for longer than 0.5 seconds^22^. For the coding, we selected the laugh face that was closest to the midpoint of each play bout as well as sufficiently visible for the FACS-based coding. In case the face was not fully visible on the specific frame, we considered the previous and the following frame to get a better view of the expression. To increase the number of chimpanzee laugh faces, we also identified the first and the last laugh face of each play bout, as long as they were at least 2 s away from the beginning/ending of the bout and from the laugh face that was already coded in the middle of the play bout. Using the aforementioned approach, we identified laugh faces belonging to both players within the same play bout, when possible.

For every laugh face, we reported its onset and offset and we then coded its midpoint, where we searched for all facial activations described in the OrangFACS^26^ and ChimpFACS manuals^34^, namely AUs (Action Units) and ADs (Action Descriptors). We excluded, however, eye closure/blink (AU43/45) and vocalisation (AU50) from the coding as they do not contribute to the laugh faces’ morphology (for an exhaustive list of the facial activations coded in this work, see Table 2). In total, we identified 302 and 322 laugh faces for the orangutans and for the chimpanzees, respectively. OrangFACS^20^ and ChimpFACS^21^ coding was conducted by FC, a certified coder for both.

**Table 2:** AU/AD percent occurrence in the orangutan and chimpanzee subjects.

		Percent occurrence	
		Orangutans	Chimpanzees
AU6	Cheek raise	1.66	1.56
AU9	Nose wrinkle	0.99	0
**AU10**	**Upper lip raiser**	**65.45**	**25.78**
**AU12**	**Lip corner puller**	**6.95**	**13.35**
**AU16**	**Lower lip depressor**	**64.33**	**75.47**
AU17	Chin raise	0.33	0
AU18	Lip pucker	0.33	0
AU22	Lip funnel	0.99	0.62
AU24	Lip press	1.99	0
**AU27**	**Mouth stretch**	**74.17**	**44.72**
AD19	Tongue protrusion	5.30	0
AD160	Lower lip relaxation	1.32	9.63

The list includes all measured AUs/ADs besides the always present AU25 as well as AU26. As AU26 and AU27 are mutually exclusive, only the latter was reported here and further analysed. The four AUs most frequently observed are marked in bold. AUs not contributing to the facial morphology (AU43/45, AU50) were not assessed in this study.

The facial orientation (whether the subject and playmate faced each other within 45 degrees of head rotation^56^, the subject biting behaviour (whether the subject bit the playmate 1 s before/after the coded expression), and the subject play action and play intensity were also coded. Play actions in this study were 10 mutually-exclusive behaviours, grouped into three levels of intensity (according to Davila-Ross and colleagues^25,55^): low (slow grappling, tickling, invitation to play, approaching), medium (fast grappling, gnawing, wrestling), and high (chasing, jumping, hitting).

To examine social relationships, we measured social proximity (for both orangutans and chimpanzees)^57–59^ as well as grooming (for chimpanzees only; the orangutans of this study did not show this behaviour), by using the focal recordings only. For social proximity, we identified the conspecifics that were within 1-m proximity of the subject, by using a single sampling instance at the beginning of the recording. In total, 923 orangutan recordings were used (30 to 129 per focal individual; mean = 65.93; SD = 29.46), and 930 chimpanzee recordings (40 to 50 per focal individual; mean = 44.29; SD = 5.07). For grooming, we identified chimpanzees involved in grooming, regardless of its direction (grooming the focal individual or vice versa).

Behavioural coding was performed with Solomon Coder software (version beta 19.08.02; retrieved from https://solomon.andraspeter.com/). Inter-coder reliability tests were conducted based on 15% of the videos using Cohen’s Kappa agreement^60^ and resulted in substantial agreement (orangutans: play actions/intensity: k = 0.77, facing: k = 0.63; chimpanzees: play actions/intensity: k = 0.79, facing: k = 0.72).

### Calculations and statistical analyses

For social relationships, we used the social proximity and grooming data to calculate social bond strength using the dyadic sociality index (DSI) for each dyad^61^, an index widely used in primate research^62–64^. As such, we followed the formula below, where *d* is the number of behaviours considered, *f*_*ixy*_ is the rate of the behaviour *I* for the dyad *xy*, and *f*_*i*_ is the mean rate of the behaviour *i* across all dyads:

$$DSI_{xy} = \frac{{\sum\nolimits_{i = 1}^{d} {\frac{{f_{ixy} }}{{f_{i} }}} }}{d}.$$

We tested our predictions with a series of Generalised Linear Mixed Models (GLMMs) with Binomial distribution and Logit function using R software (version 4.1.2). To compute the models we used the function “glmer” from the package lme4^65^. We generated four models per taxon, one for each AU of interest (AU10, AU12, AU16 and AU27). For each model, we assigned the AU of interest (presence/absence) as the response factor with subject and playmate identity as random factors. We included a total of six predictors in this study: sex of playmate, age difference (“subject age” minus “playmate age”, in years), and dyadic social index (DSI), as well as play intensity (low, medium or high), biting (present immediately before/after the coded laugh face, or absent), and playmate facing the subject (present or absent).

Since the orangutans of this study often were in close contact when they played, while placing their faces near to the other’s body, it was difficult to code for biting and facing on several occasions. Specifically, biting could not be coded for 80 instances (26.5% of the total, due to a lack of visibility in the videos), so that the GLMM analysis was carried out twice for this taxon, i.e., with the complete data set excluding biting and with the reduced data set including biting. Furthermore, facing could not be coded in over half of the instances (59.6% of the total, due to a lack of visibility in the videos), so that it was not considered to be a reliable predictor and had to be excluded from all models of this taxon.

If age difference or sex of the playmate were significant predictors of AU activation, we examined the interaction between “age difference:sex of the playmate” in an additional model. Such interaction was not included in the first models since it was then too complex to converge in some cases.

## Results

In total, we found 14 distinctive AUs/ADs present in orangutan laugh faces and 9 in chimpanzee laugh faces, including the two AUs defining a laugh face (i.e., lips parting via AU25 and jaw dropping/stretching via AU26/27). The four AUs that were found to occur most frequently were AU10, AU12, AU16, and AU27 for both taxa; these AUs were further examined with GLMMs. For a complete list of the remaining AUs/ADs, see Table 2.

### Orangutan laugh faces

For the orangutans, AU10 (upper lip raiser) was significantly more likely to occur the younger the playmates were compared to the subjects (p = 0.015). When including the interaction “age difference:sex of the playmate”, we found that AU10 in orangutans was significantly more likely to occur the younger the male playmates were compared to the subjects (p = 0.005). AU12 (lip corner puller) tended to be more often present when the playmates were females (p = 0.054). AU16 (lower lip depressor) tended to be more frequent in high intensity play compared to less intense play (p = 0.059). Furthermore, the GLMM analysis conducted on the subsample that included “biting” showed that AU16 was significantly more likely to occur when the subjects were biting their playmates (p = 0.028). AU27 (mouth stretch) was significantly more likely to occur in high intensity play compared to less intense play (p = 0.036). All other GLMM outcomes for these AUs were not found to be statistically significant (for further GLMM results, see Table 3).

**Table 3:** Orangutan GLMM analysis for the four AUs of interest.

Orangutans main models					
	Fixed effect	Estimated	Std. error	z-value	p-value
AU10	Intercept	0.092	0.710	0.130	0.897
	Age difference	0.130	0.053	2.425	0.015*
	Playmate sex	− 0.146	0.400	− 0.371	0.711
	Social relationship	0.045	0.111	0.403	0.687
	Play intensity	0.447	0.302	1.481	0.139
	Biting	− 0.192	0.334	− 0.575	0.565
(post-hoc analysis)	Age difference: playmate sex	0.179	0.064	2.806	0.005*
AU12	Intercept	− 3.364	1.065	− 3.159	0.002
	Age difference	0.091	0.055	1.637	0.102
	Playmate sex	− 0.945	0.490	− 1.928	0.054
	Social relationship	− 0.058	0.155	− 0.375	0.708
	Play intensity	0.719	0.477	1.506	0.132
	Biting	− 1.098	0.791	− 1.388	0.165
AU16	Intercept	− 0.417	0.668	− 0.624	0.533
	Age difference	0.036	0.048	0.748	0.455
	Playmate sex	0.059	0.436	0.135	0.893
	Social relationship	− 0.083	0.100	− 0.829	0.407
	Play intensity	0.540	0.286	1.886	0.059
	Biting	0.725	0.329	2.200	0.028*
AU27	Intercept	− 0.304	0.672	− 0.453	0.651
	Age difference	− 0.011	0.047	− 0.226	0.821
	Playmate sex	0.168	0.363	0.461	0.645
	Social relationship	− 0.073	0.111	− 0.657	0.512
	Play intensity	0.650	0.309	2.103	0.036*
	Biting	0.048	0.361	0.133	0.894

“Biting” was included in a separate model due to the high incidence of missing data for this predictor. Significant results are marked with an asterisk.

### Chimpanzee laugh faces

For the chimpanzees, AU10 (upper lip raiser) was significantly more likely to occur the older the playmates were compared to the subjects (p = 0.027) and in medium/high intensity play compared to low intensity play (p = 0.016 and p = 0.006, respectively). When including the interaction “age difference:sex of the playmate”, we found that AU10 in chimpanzees was significantly more likely to occur the older the female playmates were compared to the subjects (p = 0.022). AU12 (lip corner puller) was significantly more likely to occur in high intensity play compared to lower intensity play (p = 0.001). AU16 (lower lip depressor) was significantly more likely to occur the older the playmates were compared to the subjects (p < 0.001), when the players were interacting in medium/high intensity play compared to low intensity play (p = 0.012 and p = 0.005, respectively), and when the players were facing each other (p = 0.007). When including the interaction “age difference:sex of the playmate”, we found that AU16 in chimpanzees was significantly more likely to occur the older the female playmates were compared to the subjects (p < 0.001). AU27 (mouth stretch) was significantly more present in medium and high intensity play compared to low intensity play (p = 0.012 and p = 0.001, respectively), and when the subjects were biting their playmates (p = 0.014) (for further GLMM results, see Table 4).

**Table 4:** Chimpanzee GLMM analysis for the four AUs of interest.

Chimpanzees main models					
	Fixed effect	Estimated	Std. error	z-value	p-value
AU10	Intercept	− 2.572	0.696	− 3.697	< 0.001
	Age difference	− 0.034	0.015	− 2.218	0.027*
	Playmate sex	− 0.008	0.332	− 0.025	0.980
	Social relationship	− 0.033	0.052	− 0.624	0.532
	Play intensity	0.821	0.296	2.773	0.006*
	Biting	0.024	0.342	0.069	0.945
	Facing	− 0.457	0.273	− 1.671	0.095
(post-hoc analysis)	Age difference:playmate sex	− 0.087	0.038	− 2.295	0.022*
AU12	Intercept	− 4.170	0.822	− 5.072	< 0.001
	Age difference	− 0.027	0.019	− 1.397	0.162
	Playmate sex	− 0.450	0.414	− 1.086	0.278
	Social relationship	0.062	0.052	1.183	0.237
	Play intensity	1.124	0.350	3.210	0.001*
	Biting	− 0.141	0.451	− 0.312	0.755
	Facing	− 0.193	0.341	− 0.566	0.572
AU16	Intercept	− 0.907	0.706	− 1.284	0.199
	Age difference	− 0.055	0.015	− 3.709	< 0.001*
	Playmate sex	− 0.378	0.300	− 1.260	0.208
	Social relationship	− 0.004	0.050	− 0.089	0.929
	Play intensity	0.918	0.329	2.795	0.005*
	Biting	− 0.197	0.341	− 0.578	0.563
	Facing	0.774	0.287	2.701	0.007*
(post-hoc analysis)	Age difference:playmate sex	− 0.118	0.025	− 4.643	< 0.001*
AU27	Intercept	− 2.059	0.611	− 3.372	< 0.001
	Age difference	− 0.011	0.012	− 0.904	0.366
	Playmate sex	0.017	0.290	0.058	0.953
	Social relationship	0.020	0.043	0.465	0.642
	Play intensity	0.875	0.271	3.232	0.001*
	Biting	0.741	0.302	2.451	0.014*
	Facing	− 0.249	0.236	− 1.056	0.291

Significant results are marked with an asterisk.

### Sum of AUs

Furthermore, we ran a GLMM analysis per taxon with Poisson distribution and Log function, using the sum of the AUs that might contribute to the teeth baring in laugh faces as response variable (for their occurrences, see Table 2). Specifically, we included the four predominant AUs as well as AUs that may contribute to the upper teeth exposure, i.e., AU6 (cheek raise) and AU9 (nose wrinkle), and to the lower teeth exposure, i.e., AD160 (lower lip relaxation); the data showed for both taxa that every time AU6 or AU9 was coded, AU10 was also found to be present. We kept the same predictors and random factors as for the previous GLMMs. For both taxa, the resulting models showed no significant contributions of the AUs considered here (see supplementary Table 1a and b).

### Testing for potential confounds: play intensity

Of the three GLMM models that resulted in AUs occurring differently with dependence on playmate characteristics, two models also showed this pattern for play intensity: AU10 and AU16 models in chimpanzees. We, thus, controlled for play intensity via building a model that included play intensity as a random factor. The results on playmate characteristics remained unchanged (see Table 5).

**Table 5:** Chimpanzee GLMM analysis with play intensity as a random factor for AU10 and AU16.

Chimpanzees model (with play intensity as a random factor)					
	Fixed effect	Estimated	Std. error	z-value	p-value
AU10	Intercept	− 1.157	0.575	− 2.011	0.044
	Age difference	− 0.035	0.015	− 2.259	0.024*
	Playmate sex	− 0.001	0.331	− 0.003	0.998
	Social relationship	− 0.031	0.053	− 0.581	0.561
	Biting	− 0.045	0.346	− 0.130	0.896
	Facing	− 0.397	0.276	− 1.435	0.151
(post-hoc analysis)	Age difference:playmate sex	− 0.087	0.038	− 2.295	0.025*
AU16	Intercept	0.825	0.428	1.927	0.054
	Age difference	− 0.056	0.016	− 3.527	< 0.001*
	Playmate sex	− 0.419	0.340	− 1.232	0.218
	Social relationship	− 0.009	0.052	− 0.178	0.859
	Biting	− 0.192	0.347	− 0.554	0.580
	Facing	0.780	0.294	2.654	0.008*
(post-hoc analysis)	Age difference:playmate sex	− 0.118	0.025	− 4.643	< 0.001*

Significant results are marked with an asterisk.

We also compared male and female playmates for play intensity. When looking at the individual playmates, our analyses show that the orangutans played more intensively with males vs. females (Mann–Whitney U test: U = 62.0, z = 2.533, p = 0.011), while this trend was not present among the chimpanzees (Mann–Whitney U test: U = 43.5, z = 0.315, p = 0.753). The same results were also found when we looked at the playmate’s sex in all the observed dyads: orangutans (Mann–Whitney U test: U = 1835.5, z = 2.669, p = 0.008); chimpanzees (Mann–Whitney U test: U = 625.5, z = 0.031, p = 0.975).

## Discussion

The current study examined the variation in facial muscle activations in orangutans and chimpanzees during play, with a focus on the effects of playmate characteristics. We hypothesised that great apes modify their laugh face morphology with dependence on their playmate’s sex as well as their age difference and social relationship. We then predicted that they may produce some of the predominant laugh face AUs (AU10, AU12, AU16, AU27) more often when their playmates are stronger and socially more distanced in order to lower the risk of escalation into real fight (Prediction 1) or more often when the playmates are weaker in order to prevent their withdrawal and when they are socially closer individuals in order to keep the play session running (Prediction 2).

Our findings for both orangutans and chimpanzees reveal a pattern of occurrence of specific AUs that were all relevant for teeth exposure. Specifically, our orangutans produced AU10 (upper lip raiser) significantly more often when interacting with younger playmates, and they tended to show AU12 (lip corner puller) more often when interacting with female playmates. These AUs are relevant for teeth exposure, with the former exposing the upper row, and the latter enhancing its effect. In contrast, our chimpanzees produced AU10 (upper lip raiser) and AU16 (lower lip depressor) significantly more often with older individuals. These AUs are relevant for the upper teeth exposure and lower teeth exposure, respectively. Before discussing these results in relation to the literature, however, we need to consider the relative weight of potential confounds.

Previous findings have shown that play intensity, biting behaviour and facing the playmate could each affect the morphology of great ape laugh faces by enhancing the activation of facial muscles^30,40,44,45,51,52^. Regarding play intensity, our results revealed a positive association with facial muscle activations in both examined taxa, although it seems unlikely that this effect confounds the main findings of this study. In our chimpanzee models, play intensity was always among the significant predictors alongside the ones relevant to our hypothesis (in AU10 and AU16 models). However, the predictors relevant to our hypothesis were still significant even after controlling for this variable. Therefore, play intensity is unlikely to represent a valid confound for our chimpanzee results. In our orangutan models, play intensity was never co-occurring with the variables that emerged as significant predictors for our hypothesis. Consequently, play intensity is also unlikely to be a confounding factor for the orangutan results.

It is interesting to note that our orangutan subjects opened their mouths more widely (AU27) and tended to expose the lower teeth (AU16) more frequently in rough play. Similarly, our chimpanzee subjects used all four predominant AUs (AU10, AU12, AU16, AU27) more frequently as the play became rougher. These results are in line with previous findings. Gorillas and bonobos were found to expose their upper teeth more often in these contexts^30,40,44^, while orangutans seem to stretch (rather than just open) their mouths more often in more intense play^45^. All great ape taxa were also found to produce more laughter/laugh faces in more risky play, i.e. rough and contact play vs. gentle and non-contact^30–34,58,66^. Similarly, human infants may produce laugh faces with their mouths more widely opened and their teeth more exposed when they are involved in physically engaging play^17^, and pre-school children seem to produce their laugh faces with teeth exposure predominantly when social play is rough (and laugh faces without teeth exposure when it is gentle)^18^.

Biting behaviour, which has previously been linked to some AUs, such as the ones responsible for the mouth opening and the teeth-baring^51,52^, was in the current study a significant predictor only once per taxon, i.e., for the lower teeth exposure (AU16) in orangutans and for the wide mouth stretch (AU27) in chimpanzees. It is important to note, however, that neither of these two models included significant predictor to our hypothesis, therefore biting is not likely to be a potential confound. Furthermore, facing the partner while producing a laugh face, was only a significant predictor for AU16 (lower lip depressor) in chimpanzees. Specifically, the chimpanzee subjects were found to produce laugh faces with lower teeth exposure more often when facing their playmates than when not facing them. Therefore, although it is unlikely that facing the playmate is an important confound for the other chimpanzee models, it might represent a possible confound for the lower teeth exposure. Notably, this variable has not been included in the orangutan models due to the high amount of missing data. It is therefore not possible to discuss the impact of facing in this group without more extensive analyses.

Overall, our results suggest that while the orangutans of this study predominantly exposed the upper teeth when playing with weaker individuals, possibly to reassure the social partner, the chimpanzees of this study predominantly exposed both teeth rows when playing with stronger playmates, possibly signalling submissiveness. This seems to align with previous findings on primates. Similar to orangutans, adult gelada monkeys were found to expose their teeth more often than their subadults in mixed-age dyads^42^. Contrastingly, juvenile chimpanzees, when playing with younger individuals, are not more likely to produce more laugh faces than their counterparts^32^. A similar pattern is observed in our species too. Adult human males gather more smiles than females^9,10^ especially when older^11–14^, showing that, in both humans and chimpanzees, the tooth row exposures occur more frequently towards stronger rather than weaker individuals.

The opposite trends in laugh face variants observed in the orangutans and chimpanzees of this study might be explained based on their evolutionary history, their social and ecological environment and rearing history. Due to their hierarchical social structure, it might be important for chimpanzees to effectively communicate non-hostility towards individuals who could harm them, hence favouring communication directed to potentially threatening individuals. It is therefore plausible that a signal for playfulness evolved closely linked to the chimpanzee lineage to limit the number of potentially risky interactions in social play.

Orangutans, on the other hand, show solitary tendencies with most social interactions in the wild occurring between the mother and her offspring^67^, which rely on this parent for acquiring their survival skills. Consequently, in this taxon, it might be more important to focus on the communication proceeding from the older individual towards the younger individual. This interpretation, however, could only partially explain our orangutan results, as it does not clarify why adult males and juveniles of both sexes should adopt such a strategy. Thus, it seems at least partly unclear why such signals of playfulness promote interaction with younger (and potentially less experienced) individuals in orangutans.

However, the consideration of environmental influences and the rearing history can further contribute to the discussion on the taxon-based differences in the current work. The orangutan subjects were almost exclusively orphans while the chimpanzee subjects were mostly raised by their mother in the sanctuary. The presence of maternal care is known foster the production of laugh faces in play of peers^32^. These factors, in conjunction with the fact that several individuals involved in the study have been human-reared for at least a period of time, add further caution when interpreting these results from the species’ perspective, and we do not recommend taking our findings as generalisation to orangutans and chimpanzees as a whole. Further studies on wider and more balanced samples (in terms of age and sex composition) could clarify those aspects that could not have been thoroughly investigated here, ideally providing a better view of great apes’ facial expressions’ functions.

When considering the interaction of age difference and playmate sex, we found an unexpected trend in the orangutan data, with their upper teeth (via AU10) more frequently directed towards younger males compared to females. We also found an unexpected trend for the chimpanzees, i.e., exposing both their tooth rows (via AU10 and AU16) when playing with older females compared to males. Since play intensity and biting do not seem to be confounding variables for both taxa in this study, the explanation for these trends resides elsewhere, possibly in the composition of our sample. In chimpanzees, for example, the male–female ratio among the seven youngest playmates was unbalanced, with only one male (an infant). Collectively, these unexpected trends suggest that this topic requires notably more attention and that laugh communication is notably more complex in function than currently perceived.

Our findings on the social relationships did not show any indication of AU modifications in any of the models. Similarly, other studies on monkeys found no effect of social bonds on facial expressions variants^41^. Among great apes, bonobos tend to produce more laugh faces in dyads that include individuals of similar ranks^43^, and lowland gorillas mimic the playmate’s laugh face more often in dyads that include more socially distant individuals^68^. Humans also tend to produce more expressions of positive emotions when with friends compared to strangers, both as adults^8^ and as infants^16^. As we did not take into consideration variables such as the individuals’ rank or the rate of mimicry, our results should not be considered in open contrast with the pre-existing literature due to the different methodological approaches.

Our results on the total number of AUs contributing to the laugh face’s production also did not corroborate the literature. While previous studies have shown that apes produce more laugh faces when the recipient is facing the subject (great apes^31,45,69^; lesser apes^70,71^), facing the playmate was not a significant predictor in our models, with the exception of the lower teeth exposure (via AU16) in chimpanzees. The work from Waller and colleagues^45^ in particular, found that young (3-12yo) Bornean orangutans use more marked expressions (with the mouth stretched rather than just opened) when facing the playmate, and that the number of AUs is negatively correlated to the intensity of the play (ibid.). Notably, an important difference between our study and the work of Waller and colleagues is that we did not measure the frequency of laugh faces throughout the play bouts, coding instead the laugh faces displayed in a specific moment.

In conclusion, our findings showed that nonhuman great apes produce morphologically distinct laugh face variants depending on the playmate’s characteristics, specifically regarding their age and sex. Our results are, thus, supporting our hypothesis, i.e., that great apes modify their facial expression’s morphology depending on the playmate’s characteristics. These findings are building on previous research showing that nonhuman primates adjust their facial expressions, in general, depending on the social circumstances^32,41,45^. Overall, this work highlights the complex use of laugh faces in great apes. Furthermore, it suggests that laugh faces already served multiple social functions in ancestral apes and that such complexity in form and function further evolved as laugh faces became the highly pervasive tools of human social communication we observe nowadays.

## Supplementary Information

Supplementary Table 1.

## Data Availability

The raw data used for this study will be made available by the corresponding authors, without undue reservation.
