# Supplementary material for: Orangutans and chimpanzees produce morphologically varied laugh faces in response to the age and sex of their social partners
Source: Sci Rep. 2024 Nov 6;14:26921. doi: 10.1038/s41598-024-74089-x (PMC11542029; doi:10.1038/s41598-024-74089-x)
Supplement: Supplementary file 1 — Supplementary Table 1. [file 41598_2024_74089_MOESM1_ESM.docx]

**SUPPLEMENTARY MATERIAL**

**Supplementary table 1a.** Orangutan GLMM analysis for the sum of AUs.

| **ORANGUTANS SUM OF AUs** | |  |  |  |  |
| --- | --- | --- | --- | --- | --- |
|  | **fixed effect** | **estimated** | **std. error** | **z-value** | **p-value** |
| Sum of AUs | intercept | 1.110 | 0.143 | 7.772 | <0.001 |
|  | age difference | 0.011 | 0.008 | 1.481 | 0.139 |
|  | playmate sex | -0.002 | 0.075 | -0.026 | 0.979 |
|  | social bond | 0.008 | 0.017 | 0.484 | 0.628 |
|  | play intensity | 0.063 | 0.065 | 0.966 | 0.334 |

**Supplementary table 1b.** Chimpanzee GLMM analysis for the sum of AUs.

| **CHIMPANZEES SUM OF AUs** | |  |  |  |  |
| --- | --- | --- | --- | --- | --- |
|  | **fixed effect** | **estimated** | **std. error** | **z-value** | **p-value** |
| Sum of AUs | intercept | 0.965 | 0.146 | 6.602 | <0.001 |
|  | age difference | -0.003 | 0.003 | -0.958 | 0.338 |
|  | playmate sex | -0.040 | 0.071 | -0.565 | 0.572 |
|  | social bond | 0.001 | 0.011 | 0.111 | 0.912 |
|  | play intensity | 0.115 | 0.064 | 1.782 | 0.075 |
|  | biting | -0.022 | 0.081 | -0.266 | 0.791 |
|  | facing | -0.013 | 0.062 | -0.216 | 0.829 |
